# Emergence of IncX3 Plasmid-Harboring *bla*_NDM–5_ Dominated by *Escherichia coli* ST48 in a Goose Farm in Jiangsu, China

**DOI:** 10.3389/fmicb.2019.02002

**Published:** 2019-09-04

**Authors:** Ziyi Liu, Xia Xiao, Yan Li, Yuan Liu, Ruichao Li, Zhiqiang Wang

**Affiliations:** ^1^College of Veterinary Medicine, Yangzhou University, Yangzhou, China; ^2^Jiangsu Co-innovation Center for Prevention and Control of Important Animal Infectious Diseases and Zoonoses, Yangzhou, China; ^3^Institute of Comparative Medicine, Yangzhou University, Yangzhou, China; ^4^Institutes of Agricultural Science and Technology Development, Yangzhou, China

**Keywords:** carbapenemase genes, *bla*_NDM–5_, *E. coli*, long-read sequencing, plasmids

## Abstract

Twelve carbapenem-resistant *Escherichia coli* strains were obtained from goose farms in Jiangsu, China. These isolates were resistant to multiple antimicrobials, and positive for the *bla*_NDM–__5_. The carbapenem-resistance of all strains mediated by *bla*_NDM–__5_ were successfully conjugated to *E. coli* J53. S1-PFGE and WGS results showed *bla*_NDM–__5_ was located on IncX3 conjugative plasmids with a size of ca. 46 kb. All *bla*_NDM–__5_-bearing IncX3 plasmids shared the same genetic context almost identical to pNDM_MGR194-*bla*_NDM–__5_ and pNDM-QD28-*bla*_NDM–__5_ reported in India and China, respectively. The twelve strains belonged to three STs, in which the dominant type of *E. coli* isolated from breeding goose farm carrying *bla*_NDM–__5_ was ST48. The emergence of *bla*_NDM–__5_-bearing strains in goose farms and the clonal transmission of *E. coli* within the breeding goose farm highlighted the potential reservoir of carbapenemase genes in waterfowl farming system, which may further contaminate environments and pose a threat to public health. Comprehensive surveillance of carbapenem-resistant bacteria in goose farms warrants further study to evaluate the underlying risks.

## Introduction

*Enterobacteriaceae* such as *Escherichia coli*, *Klebsiella pneumoniae* and *Salmonella* spp. are important pathogens that cause human infections. Carbapenemase-producing *Enterobacteriaceae* (CPE) is constantly reported worldwide and has become an urgent public health threat (Potter et al., 2016). So far, CPE has been detected in animals, environment and vegetable samples that are closely related to humans (Walsh et al., 2011; Wang J. et al., 2018; Wang R.B. et al., 2018). As one main type of carbapenemases, New Delhi metallo-β-lactamase (NDM) is able to confer resistance to almost all β-lactams. Since the first report of *bla*_NDM–__1_ in 2009, 21 variants of NDM enzymes (NDM-1 to NDM-21) have been reported (Yong et al., 2009; Liu et al., 2018). Among them, the NDM-5-encoding gene, *bla*_NDM–__5_, was first reported in an *E. coli* recovered from a patient in the United Kingdom (Hornsey et al., 2011). In China, a lot of studies have reported the *bla*_NDM–__5_ in *Enterobacteriaceae* of animal origin. Regarding poultry origin, there was one report of the presence of NDM in *Enterobacteriaceae* in the poultry production environment, which may indicated that *bla*_NDM–__5_ has been widely distributed among *Enterobacteriaceae* in poultry farms (Zhang et al., 2019). Especially, the coexistence of *bla*_NDM–__5_ and *mcr-1* in *E. coli* of single duck origin has also been reported, which raised the public concern about the antibiotic resistance of waterfowl (Yang et al., 2016).

In addition to duck origin, goose breeding industry is particularly developed in Jiangsu, China. The breeding mode of geese in farms is different from other poultry farms, because many pools are built in goose farms to support their growth. Owing to the use of antibiotics and possible dissemination of resistant bacteria via water system in goose farms, the antibiotic resistance may be extremely complicated. However, there are few reports of antibiotic resistance in goose farms. Therefore, enhancing the surveillance of antibiotic resistance in goose farms is necessary. To cover this gap, we investigated the prevalence of *bla*_NDM–__5_-positive *E. coli* isolates in goose farms and probed the genomic features of these isolates with cutting-edge nanopore long-read sequencing technology.

## Materials and Methods

### Identification of Bacterial Strains and Detection of Carbapenemase Genes

A total of 117 samples, including anal swab, feed, feces, water and soil, were collected within one day from a breeding goose farm (*n* = 68) and a goose hatchery (*n* = 49) in Jiangsu Province, China in 2018 (Table S1). There are about 10 goose houses in the breeding farm where farming scale approaches to one thousand. We randomly selected three goose houses as sample sites. It was worth pointing out that we considered the samples collected from the three goose houses as related samples due to the same feed and water used in different goose houses, the open environment and the free movement of the breeders. The hatchery farm which isolated from outside had some distance away from the breeding goose farm (Figure S7). Both farms belonged to the same goose producing chain meaning that the goslings from hatchery were transported to the breeding farm for feeding. To be note, the breeding goose were mainly for food and the water supply of the goose farm was from the river outside the farm. Besides, there were no hospitals, factories or other source of pollutions near the farm. Isolation of carbapenem-resistant *E. coli* was performed as following method. Briefly, each sample was added to 3 mL of buffer peptone water (BPW) and incubated for 6–8 h in 37°C, followed by inoculating into Mossel Enterobacteria enrichment broth (MEE broth) with 0.5 mg/L meropenem for 6 h. The carbapenem-resistant bacteria were isolated by streaking on MacConkey plates containing 2 mg/L meropenem and identified by MALDI-TOF MS and 16S rDNA sequencing (Kim et al., 2010). In this way, significant number of well-formed carbapenem-resistant colonies were grown on MacConkey plates, but only one colony was selected for further investigations. The carbapenem-resistant isolates were screened for the most found β-lactamases genes including the OXA-1-like broad-spectrum β-lactamases, extended-spectrum β-lactamases (ESBLs), plasmid-mediated AmpC β-lactamases and class A, B and D carbapenemases using multiplex PCR assays reported previously (Dallenne et al., 2010). The complete coding sequence of *bla*_NDM–__5_ was amplified with the primers (NDM-up, 5′-CTTCCAACGGTTTGATCGTC; NDM-dw, 5′-ATTGGCATAAGTCGCAATCC) and confirmed by ABI3730 sequencing.

### Antimicrobial Susceptibility Testing, Conjugation Assay and S1-PFGE

The MICs of *bla*_NDM–__5_-positive *E. coli* isolates against antibiotics (Table 1), as well as their transconjugants, were determined using the microdilution broth method and interpreted according to CLSI (2014) guidelines. *E. coli* strain ATCC 25922 was used as the quality control. To investigate transferability of *bla*_NDM–__5_, conjugation assay was performed for the twelve *bla*_NDM–__5_ positive *E. coli* isolates with the sodium azide-resistant *E. coli* J53 as the recipient strain. Overnight culture of donor strains and *E. coli* J53 were mixed (ratio of 1:4) in LB broth, then subjected to overnight incubation on LB agar plates. The mixture culture was then diluted and spread on a selective LB agar plate supplemented with meropenem (2 mg/L) and sodium azide (200 mg/L) to recover transconjugants. Carriage of *bla*_NDM–__5_ in the transconjugant was confirmed by PCR and MICs. The frequencies of conjugation transfer of five *bla*_NDM–__5_ plasmids were conducted and expressed as transconjugants per donor cell (T/D) as previously described (Zhong et al., 2012). S1-PFGE was performed to obtain plasmid profiles in donor strains and transconjugants, and the *Salmonella enterica* serotype Braenderup H9812 was used as the standard size marker.

**TABLE 1:** Antibiotic susceptibility profiles of the carbapenem-resistant *E. coli* strains and the corresponding transconjugants to different antibiotics (mg/L).

**Isolates**	**Antibiotics^a^**								
	**MEM**	**STR**	**ATM**	**AMX**	**CQM**	**FFC**	**DOX**	**ENR**	**CL**
L33	128	128	256	>64	>128	>64	32	16	1
L37	128	64	32	>64	>128	>64	16	16	1
L41-1	128	16	4	>64	>128	>64	32	≤0.125	1
L43-1	256	32	8	>64	>128	>64	32	0.25	1
L53	128	128	256	>64	>128	>64	32	4	1
L56	128	>128	>256	>64	>128	>64	16	8	1
L65	128	1	≤ 0.5	>64	>128	>64	32	1	1
L99	128	>128	>256	>64	>128	>64	32	4	1
L100	128	64	32	>64	>128	>64	32	≤0.125	0.5
L102	128	32	8	>64	128	4	16	≤0.125	1
L103-1	64	128	>256	>64	>128	>64	16	8	0.125
L103-2	128	64	256	>64	>128	>64	16	≤0.125	1
L33T	128	1	≤0.5	>64	128	8	4	≤0.125	0.5
L37T	32	1	≤0.5	>64	128	8	4	≤0.125	0.5
L41-1T	64	0.5	2	>64	>128	4	8	≤0.125	0.5
L43-1T	64	1	1	>64	>128	8	8	≤0.125	0.25
L53T	128	1	64	>64	128	8	4	≤0.125	1
L56T	64	1	≤0.5	>64	128	4	4	≤0.125	0.5
L65T	64	1	≤0.5	>64	128	4	2	≤ 0.25	0.5
L99T	64	2	≤ 0.5	>64	64	4	2	≤ 0.125	1
L100T	64	1	2	>64	128	4	2	≤0.125	1
L102T	128	1	1	>64	>128	4	4	≤ 0.125	0.5
L103-1T	128	1	≤ 0.5	>64	128	8	4	≤ 0.125	1
L103-2T	64	1	64	>64	128	4	8	≤0.125	1
J53^b^	≤0.25	1	≤0.5	1	≤0.25	4	4	≤0.125	0.5
ATCC25922^c^	≤0.25	0.5	≤0.5	1	≤0.25	2	0.5	≤0.125	0.5

*^*a*^MEM, meropenem; STR, streptomycin; ATM, aztreonam; AMX, amoxicillin; CQM, cefquinome; FFC, florfenicol; DOX, doxycycline; ENR, enrofloxacin; CL, colistin. All susceptibility tests were repeated at least three times according to CLSI method. The results of colistin susceptibility were interpreted according to EUCAST breakpoints. ^*b*^recipient strain. ^*c*^quality control strain.*

### DNA Extractions, WGS and Bioinformatics Analysis

Genomic DNA of the twelve carbapenem-resistant strains were prepared using the TIANamp Bacteria DNA Kit (Tiangen, China) and subjected to WGS using the Illumina HiSeq 2500 platform (Illumina, San Diego, CA, United States) generating 2 × 150 bp paired-end reads (1 Gbp per sample). The software SPAdes (v3.11.1) was utilized to assemble the genomes. Furthermore, genomes of six strains (L37, L41-1, L53, L65, L100, and L103-2) belonging to different clonal groups based on Illumina data were selected for MinION long-read sequencing (400 Mbp per sample) with the Rapid Barcoding Kit RBK004 without size selection to obtain the complete genome sequences according to the published method (Li R. et al., 2018). Briefly, *de novo* assembly with hybrid strategy combining Illumina short-read data and MinION long-read data was performed with unicycler (v0.4.4) as the reported method (Wick et al., 2017; Li R. et al., 2018). Sequence Types (STs) and antimicrobial resistance genes were determined using the GoSeqIt tool^1^. Plasmid replicon type and plasmid multi-locus STs were determined using the PlasmidFinder and pMLST tools^2^. The complete genome sequences were annotated using the RAST^3^ automatically and modified manually. Comparisons between *bla*_NDM–__5_-bearing plasmids and homologous plasmid sequences available in NCBI database were performed using the BRIG tool and Easyfig (Alikhan et al., 2011; Sullivan et al., 2011). The phylogenetic tree based on SNPs was generated as previously reported (Li et al., 2017).

The complete genome sequences of the six strains were deposited in NCBI database with the accession numbers listed in Table 2. The *de novo* assembled results of another six strains were deposited in figshare database^4^ for reference.

**TABLE 2:** Basic information of the twelve *bla*_NDM–__5_ positive *E. coli* strains revealed by WGS data.

									**Number of**	
		**MLST**		**Conjugation**		**MinION**	**Plasmid names**	**Size**	**resistance**	**Accession**
**Strains**	**Species**	**types**	**Source**	**Transferability^a^**	**Resistance genes**	**sequencing**	**(or replicons)**	**(bp)**	**genes**	**numbers**
L37	*Escherichia coli*	ST-48	Anal swab	+	*fosA, sul2, aac(3)-IId, strA, strB, qnrS1, dfrA14, tet*(A)*, floR, bla*_CTX–M–55,_ *bla*_NDM–5,_ *bla*_TEM–1B,_ *aph(6)-Id, aph(3″)-Ib*	+	pL37-2 (IncFIB, p0111)	145704	9	CP034590
							pL37-3 (IncX3)^d^	45650	1	CP034591
							pL37-4 (IncFII)	76559	3	CP034592
L41-1	*Escherichia coli*	ST-48	Feed	+	*strA, strB, sul2, floR, bla*_CTX–M–64,_ *bla*_NDM–5,_ *bla*_TEM–1A,_ *tet*(A)	+	pL41-1-2 (IncFIB, IncHIB, p0111)	201021	8	CP034728
							pL41-1-3 (IncFIB)	111458	1	CP034729
							pL41-1-4 (IncX3)^d^	46238	1	CP034730
L53	*Escherichia coli*	ST-48	Water	+	*fosA, sul2, strA, strB, aac(3)-IId, qnrS1, dfrA14, tet*(A)*, floR, bla*_TEM–1B,_ *bla*_NDM–5,_ *bla*_CTX–M–55_	+	pL53-2 (IncFIB, p0111)	137848	9	CP034745
							pL53-3 (IncFII)	76642	3	CP034756
							pL53-4 (IncX3)^d^	46259	1	CP034757
L65	*Escherichia coli*	ST-3076	Feces	+	*tet*(A)*, qnrS1, dfrA14, floR, bla*_TEM–1C,_ *bla*_NDM–5_	+	pL65-2 (IncFIB, IncFII)	145346	6	CP034739
							pL65-9 (IncX3)^d^	46161	1	CP034744
L100	*Escherichia coli*	ST-48	Anal swab	+	*strA, strB, sul2, floR, bla*_CTX–M–64,_ *bla*_TEM–1A,_ *bla*_NDM–5,_ *tet*(A)	+	pL100-2 (IncFIB, IncHIB, p0111)	202387	8	CP034746
							pL100-3 (IncFIB)	111458	1	CP034747
							pL100-4 (IncX3)^d^	46261	1	CP034748
L103-2	*Escherichia coli*	ST8809	Anal swab	+	*arr-2, rmtB, aph(3′)-IIa, aadA1, fosA, dfrA14, tet*(A)*, cmlA1, floR, bla*_TEM–1B,_ *bla*_NDM–5,_ *bla*_OXA–10,_ *bla*_CTX–M–55_	+	pL103-2-2 (IncFIB)	110210	0	CP034844
							pL103-2-3 (IncFIB)	102295	1	CP034845
							pL103-2-4 (IncFII)	100667	6	CP034846
							pL103-2-5 (IncX3)^d^	46163	1	CP034847
							pL103-2-6 (IncX1)	13362	1	CP034848
							pL103-2-7 (unknown)	12606	6	CP034849
L33	*Escherichia coli*	ST-48	Anal swab	+	*strB, strA, aac(3)-IId, sul2, fosA, qnrS1, dfrA14, tet*(A), *floR, bla*_NDM–5,_ *bla*_CTX–M–55,_ *bla*_TEM–1B_	–	IncX3^d^, p0111	46kb^c^	12	Online^b^
L43-1	*Escherichia coli*	ST-48	Feces	+	*strA, strB, sul2, floR, bla_CTX–M–64,_ bla_TEM–1A,_ bla_NDM–5,_ tet*(A)	–	IncX3^d^, p0111, colRNAI	46kb	8	Online
L56	*Escherichia coli*	ST-48	Water	+	*fosA, sul2, strA, strB, aac(3)-IId, qnrS1, dfrA14, tet*(A)*, floR, bla*_TEM–1B,_ *bla*_NDM–5,_ *bla*_CTX–M–55_	–	IncX3^d^, p0111	46kb	12	Online
L99	*Escherichia coli*	ST-48	Anal swab	+	*fosA, sul2, strA, strB, aac(3)-IId, qnrS1, dfrA14, tet*(A)*, floR, bla*_NDM–5,_ *bla*_CTX–M–55,_ *bla*_TEM–1B_	–	IncX3^d^, p0111	46kb	12	Online
L102	*Escherichia coli*	ST-48	Anal swab	+	*tet*(A)*, sul2, bla*_CTX–M–64,_ *bla*_NDM–5,_ *strB, strA*	–	IncX3^d^, p0111	46kb	6	Online
L103-1	*Escherichia coli*	ST-48	Anal swab	+	*strB, strA, aac(3)-IId, sul2, fosA, qnrS1, dfrA14, tet*(A)*, floR, bla*_NDM–5,_ *bla*_TEM–1B,_ *bla*_CTX–M–55_	–	IncX3^d^, p0111, colRNAI	46kb	12	Online

*^*a*^Transference of plasmids carrying *bla*_*NDM*–__5_. ^*b*^The assembly results of six strains without complete genome sequences were deposited in figshare database for reference (https://figshare.com/articles/L33-L103-1/8044847). ^*c*^The length of *bla*_*NDM*–__5_-bearing IncX3 plasmids in samples without MinION sequencing were infered from S1-PFGE. ^*d*^*bla*_*NDM*–__5_-bearing plasmid.*

## Results and Discussion

### Characterization of Carbapenem-Resistant *E. coli* Strains

Twelve carbapenem-resistant *E. coli* strains were isolated from the 117 samples. The twelve carbapenem-resistant *E. coli* strains included seven strains (58.3%) from anal swab samples (L33, L37, L99, L100, L102, L103-1, L103-2), two strains (16.7%) from water samples (L53, L56), two isolates (16.7%) from fecal samples (L43-1, L65) and one isolate (8.3%) from a feed sample (L41-1). Among them, L65 was isolated from goose hatchery, while the other eleven strains were isolated from the breeding goose farm indicating the occurrence of carbapenem-resistant strains of breeding goose farm was more prevalent. These strains were resistant to multiple antibiotics including meropenem, streptomycin, amoxicillin, cefquinome, florfenicol and doxycycline, but most of them were susceptible to enrofloxacin (Table 1).

All the carbapenem-resistant *E. coli* strains harbored *bla*_NDM–__5_. The strains were also found to carry multiple resistance genes conferring resistance to β-lactam (*bla*_CTX–M_, *bla*_TEM–__1_, *bla*_OXA_), aminoglycoside (*strA*, *strB*, *aac(3)-IId, aph(6)-Id*, *aph(3″)-Ib*, *rmtB*) fosfomycin (*fosA*), tetracycline (*tet*(A)), phenicols (*cmlA1*, *floR*), quinolone (*qnrS1*), sulfonamide(*sul2*), rifampicin (*arr-2*) and trimethoprim (*dfrA14*) (Table 2). Moreover, the twelve *E. coli* isolates were categorized into two STs and one novel ST according to WGS data. Ten strains belonged to ST48 and one novel ST (ST8809) strain (L103-2) was isolated from the breeding goose farm, whereas only one strain L65 isolated from hatchery pertained to ST3076, which indicated that resistant strains between the two farms was unrelated phylogenetically. Two clonal groups within ST48 were identified in breeding goose farm confirmed by SNP analysis (Figure S1).

### Transferability of *bla*_NDM–__5_ Gene and the Underlying Mechanisms

Conjugation assay was performed to investigate transferability of *bla*_NDM–__5_ gene, and transconjugants were successfully recovered from twelve donor strains (Table 1). It is noteworthy that the conjugation frequencies of *bla*_NDM–__5_-bearing plasmids were stable (∼10^–6^ per donor) (Table S2). S1-PFGE demonstrated that plasmids of ca. 46 kb in size could be observed in both donor strains and recipient strains except L53T, the transconjugant of L53, in which a plasmid of ca. 120 kb was found (Figure S2). The underlying molecular mechanism warrants further study. Two donor strains can transfer two plasmids into receipt strains (L103-1 and L103-2), enabling the two transconjugants resistant to multiple antibiotics (Table 1). The fact that all *bla*_NDM–__5_-bearing transconjugants carried a ca. 46 kb plasmid indicated *bla*_NDM–__5_ was located on a conjugative plasmid with a similar structure. To probe this hypothesis, complete genomes of six strains belonging to different clonal groups were obtained through short-read and long-read sequencing methods.

**FIGURE 1:**
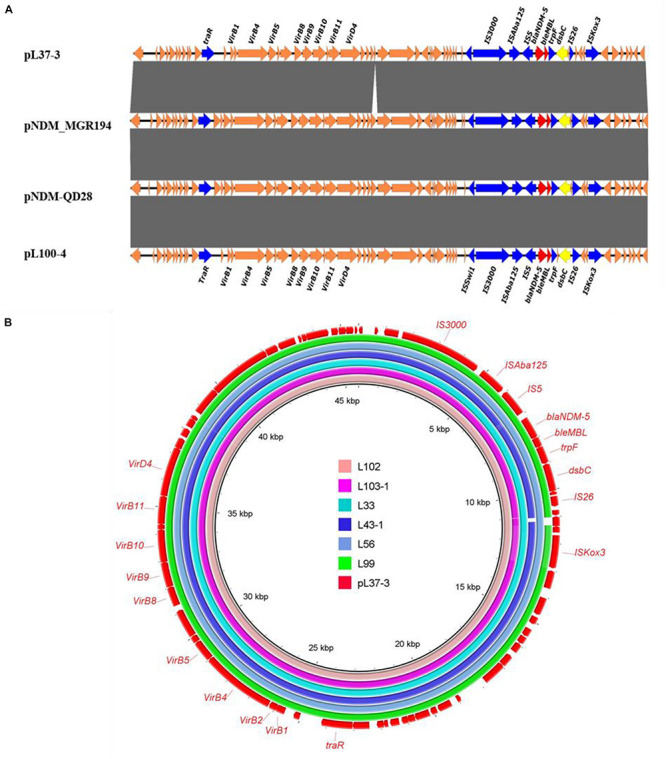
Sequence alignment analysis of IncX3 *bla*_NDM–5_ plasmids. **(A)** Linear alignment of four *bla*_NDM–5_-bearing *E. coli* strains. pNDM-QD28 (GenBank accession no. KU167608.1) and pNDM_MGR194-*bla*_NDM–5_ (GenBank accession KF220657.1) were the reported *bla*_NDM–5_-bearing plasmids. Plasmids pL41-1-4, pL53-4, pL65-9 and pL103-2-5 shown identical structure with pL100-4. **(B)** Circular comparison between the *bla*_NDM–5_-bearing IncX3 plasmid pL37-3 and the assembled contigs of other six *bla*_NDM–5_ positive strains without MinION data and based on Illumina data.

All *bla*_NDM–__5_-bearing plasmids from L37, L41-1, L53, L65, L100, L103-2 were almost identical (99% query coverage and 99% nucleotide identity) (Figure 1), with sizes ranging from 45,650 to 46,261 bp (Table 2). The *bla*_NDM–__5_ gene was in the typical structure IS*Aba125*-IS*5*-*bla*_NDM–__5_- *ble*_MBL_-*trpF*-*dsbC*-IS*26* found in IncX3 type plasmids. No other antimicrobial resistance genes were detected in these plasmids. These plasmids belonged to IncX3 type and showed 99% nucleotide identity with the first identified *bla*_NDM–__5_-harboring plasmid pNDM-MGR194 of *K. pneumoniae* MGR-K194 in India (KF220657) (Krishnaraju et al., 2015) and pNDM-QD28 obtained from *E. coli* QD28 in China (KU167608) (Figure 1). The pNDM-MGR194-like plasmids were reported in China frequently, which indicated IncX3 type plasmids were the epidemic vehicles mediating dissemination of the *bla*_NDM–__5_ in China (Krishnaraju et al., 2015; Li X. et al., 2018). The QD28 was the first identification of *bla*_NDM–__5_-carrying *E. coli* in the neonatal infection (Zhu et al., 2016). The *bla*_NDM–__5_-bearing IncX3 plasmid has been shown to be carried in several clinical isolates (Wailan et al., 2015; Zhang et al., 2016). The discovery of these *bla*_NDM–__5_-bearing plasmids in goose farm demonstrated the IncX3 plasmids accounted for widespread of *bla*_NDM–__5_ in this goose farm.

### Whole Genome Sequencing Analysis

Analysis of whole genomes of the strains revealed the distribution of antibiotic resistance genes, replicon types and STs (Table 2). The twelve strains were divided into three clonal groups based on SNP analysis, and strains belonging to ST48 from both clonal group A and C were isolated from various sources including water, anal swabs and feces collected from the breeding goose farm, demonstrating the potential clonal spread of these *bla*_NDM–__5_-bearing strains and transmission of *bla*_NDM–__5_-bearing plasmid among subtypes of ST48 in breeding goose farm (Figure S1). However, L65 (ST3076) isolated from goose hatchery and L103-2 (ST8809) obtained from breeding goose farm showed great difference from other strains (ST48) isolated from the breeding goose farm, suggesting that there was spread of *bla*_NDM–__5_ mediated by the conjugative plasmid among different *E. coli* strains (Figure S1).

Apart from the *bla*_NDM–__5_-bearing plasmids, other MDR plasmids were identified from the six complete genomes. L37 and L53 belonging to the same clonal group also carried other two plasmids, the IncFII plasmid (pL37-4, pL53-3) and the IncFIB/p0111 hybrid plasmid (pL37-2, pL53-2), respectively. A mosaic MDR region consisting of ISs and various resistance gene including *qnrS1*, *floR*, *bla*_TEM–__1__B_, *sul2*, *aac(3)-IId*, *aph(6)-Id*, *aph(3″)-Ib*, *dfrA14* and *tet*(A) was identified in IncFIB/p0111 hybrid plasmids (Figure S3). Two large similar plasmids (pL41-1-2 and pL100-2) over 200 kb were found in both L41-1 and L100 belonging to the same clonal group, and these two hybrid plasmids composing of IncFIB, IncHIB and p0111 replicons contained a MDR region (*bla*_TEM–__1__A_, *sul2*, *floR*, *tet*(A), *aph(6)-Id* and *aph(3″)-Ib*) dispersed among many insertion sequences. High insertion sequences abundance dispersed in plasmids may account for plasmid plasticity resulting in generation of hybrid plasmids. IS*26* was proved to be involved in plasmid recombination (Wong et al., 2017). The formation of these two hybrid plasmids (pL41-1-2 and pL100-2) was probably related to insertion sequences (Figures S4, S5). A hybrid plasmid pL65-2 was found in L65, and pL65-2 was composed of IncFIB and IncFII replicons and the typical characteristic of this plasmid was a transfer region containing conjugative transfer genes, but no resistance genes were found (Figure S6).

Carbapenems are currently only approved for usage in humans and prohibited in animals. The emergence of *bla*_NDM–__5_ positive *E. coli* in animals and surrounding environments posed a great public health concern. Previously, there were reports of *bla*_NDM–__5_ positive strains found in various chicken and swine farms (Ho et al., 2018; Xiang et al., 2018). However, to the best of our knowledge, this was the first report of *bla*_NDM–__5_ positive strains of goose origin which was the important waterfowl in Jiangsu breeding industry. Further research revealed that ST48 *bla*_NDM–__5_ positive *E. coli*, as dominant type, underwent clone spread within goose farm. ST48 type *E. coli* is frequently associated to various β-lactamases, including ESBL and NDM (Liu et al., 2017; Said et al., 2017), thus we assumed that goose farming system could be a reservoir of β-lactamases genes. The occurrence of carbapenem-resistant strains in goose breeding farm was higher than that in goose hatchery, which may be due to the layout of the two farms. The breeding goose farm is an open space suitable for goose activities, facilitating transmission of MDR strains. The goose hatchery adopts a completely closed layout to protect the goslings from external environments, and few MDR bacteria contaminated the goose hatchery and prevalence of resistant strains was low.

IncX3 plasmids carrying various of *bla*_NDM_ variants have been increasingly reported all over the world in recent years (Paskova et al., 2018; Xu and He, 2019). Previous study has proved the ability of *bla*_NDM_-bearing IncX3 plasmid transfer to different Enterobacterial species at a wide range of temperatures (Wang Y. et al., 2018). Based on these facts, we believed that once the IncX3 plasmid carrying *bla*_NDM–__5_ was detected in goose farms, the risk of its widespread dissemination will be greatly increased.

Water is an essential element during goose growth, and water samples were collected from the river around the goose farm. The goose farm received and discharged river water without any purification measures, which may accelerate transfer of MDR bacteria. Bacteria encoding *bla*_NDM–__5_ was found in river (Almakki et al., 2017). Thus, river water has become a reservoir of drug-resistant bacteria. Apart from the feces and feed samples, water is another environmental sample detected positive for NDM-5-producing *E. coli* in this study. This indicated that contamination of the NDM-5 producing *E. coli* between feces and water occurred in goose farm. Comprehensive surveillance of MDR bacteria in water surrounding the goose farm must be strengthened to understand the accurate contamination route. Since similar *bla*_NDM–__5_-bearing plasmids were always found in clinical settings and no carbapenem exposure existed in goose farms, this *bla*_NDM–__5_-bearing plasmid may derive from humans, further transfer and persist in goose farms to constitute a potential risk.

In conclusion, this study first identified the *bla*_NDM–__5_-bearing *E. coli* isolates in goose farms and its surroundings. Prevalence of NDM-5 producing *E. coli* posed a potential risk to public health. It is extremely urgent to reinforce the surveillance of resistance in the waterfowl system to curb the transmission or persistence of MDR bacteria.

## Data Availability

Publicly available datasets were analyzed in this study. This data can be found here: Refer to Table 2 for all accession numbers.

## Author Contributions

ZW and RL conceived and designed the study. ZL, YLi, and XX performed the study, analyzed the data, and prepared the manuscript. XX, YLiu, and RL revised the manuscript.

## Conflict of Interest Statement

The authors declare that the research was conducted in the absence of any commercial or financial relationships that could be construed as a potential conflict of interest.

## Footnotes

1
https://tools.goseqit.com/

2
http://www.genomicepidemiology.org/

3
http://rast.nmpdr.org/

4
https://figshare.com/articles/L33-L103-1/8044847
