# Corrigendum: Emergence of IncX3 Plasmid-Harboring *bla*_NDM−5_ Dominated by *Escherichia coli* ST48 in a Goose Farm in Jiangsu, China

**DOI:** 10.3389/fmicb.2022.925998

**Published:** 2022-05-12

**Authors:** Ziyi Liu, Xia Xiao, Yan Li, Yuan Liu, Ruichao Li, Zhiqiang Wang

**Affiliations:** ^1^College of Veterinary Medicine, Yangzhou University, Yangzhou, China; ^2^Jiangsu Co-innovation Center for Prevention and Control of Important Animal Infectious Diseases and Zoonoses, Yangzhou, China; ^3^Institute of Comparative Medicine, Yangzhou University, Yangzhou, China; ^4^Institutes of Agricultural Science and Technology Development, Yangzhou, China

**Keywords:** carbapenemase genes, *bla*_NDM–__5_, *E. coli*, long-read sequencing, plasmids

In the original article, we neglected to include the funder “The Postgraduate Research and Practice Innovation Program of Jiangsu Province (Yangzhou University), XKYCX18_117 to ZL.”

The authors apologize for this error and state that this does not change the scientific conclusions of the article in any way. The original article has been updated.

## Publisher's Note

All claims expressed in this article are solely those of the authors and do not necessarily represent those of their affiliated organizations, or those of the publisher, the editors and the reviewers. Any product that may be evaluated in this article, or claim that may be made by its manufacturer, is not guaranteed or endorsed by the publisher.

