# Supplementary material for: Emergence of IncX3 Plasmid-Harboring blaNDM–5 Dominated by Escherichia coli ST48 in a Goose Farm in Jiangsu, China
Source: Front Microbiol. 2019 Sep 4;10:2002. doi: 10.3389/fmicb.2019.02002 (PMC6737504; doi:10.3389/fmicb.2019.02002)
Supplement: Supplementary file 1 [file Data_Sheet_1.pdf]

Supplementary Data

Emergence of IncX3 plasmid-harboring *bla*$_{NDM-5}$ dominated by *Escherichia coli* ST48 in a goose farm in Jiangsu, China

Ziyi Liu[1,2#], Xia Xiao[1,2,3#], Yan Li[1,2], Yuan Liu[1,2,3], Ruichao Li[1,2,3]*, Zhiqiang Wang[1,2,3,4]*

[1]College of Veterinary Medicine, Yangzhou University, Yangzhou, P. R. China;

[2]Jiangsu Co-Innovation Center for Prevention and Control of Important Animal Infectious Diseases and Zoonoses, Yangzhou, P. R. China;
[3]Institute of Comparative Medicine, Yangzhou University, Yangzhou, P. R. China;
[4]Institutes of Agricultural Science and Technology Development, Yangzhou, P. R. China.

Running title： Characterization of *bla*$_{NDM-5}$-bearing goose *E. coli*

Key words: carbapenemase genes, *bla*$_{NDM-5}$, *E. coli*, long-read sequencing

[#]These authors contributed equally to the work.

*Corresponding author. Ruichao Li, Email: rchl88@yeah.net; Zhiqiang Wang, Email: zqwang@yzu.edu.cn.

Table S1. Distribution of collected samples and numbers of *bla*$_{NDM-5}$-bearing *E. coli* strains.

Farms	Sources					Total
	Anal swab	Feed	Feces	Water	Soil	
Breeding farm	7/55	1/3	1/5	2/5	0	11/68
Hatchery farm	36	2	1/2	6	3	1/49

Table S2. Conjugation frequencies of five *bla*$_{NDM-5}$-bearing IncX3 plasmids

	Isolates				
	L33	L37	L53	L65	L103-2
Conjugation frequency	2.5×10^{-6}	1.06×10^{-5}	2.5×10^{-6}	1.18×10^{-6}	8.99×10^{-6}

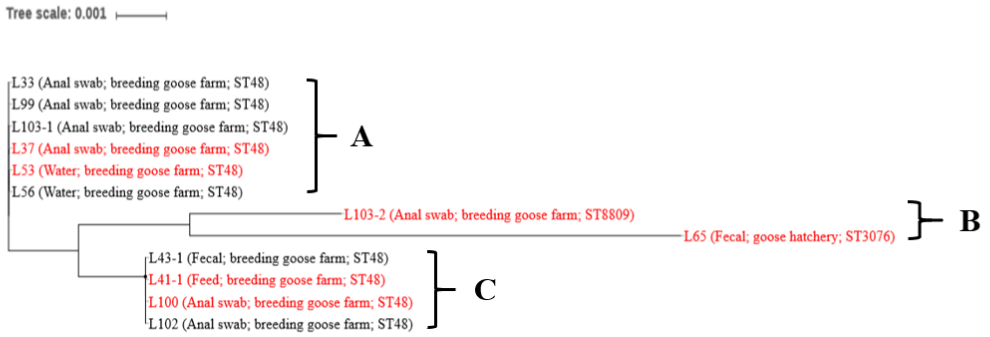

Figure S1. Maximum likelihood tree of twelve *bla*$_{NDM-5}$ positive *E. coli* based on SNPs of core genes with the roary and fasttree tools(Price et al., 2009;Page et al., 2015). Three major clonal groups (CGs) were identified. Compared with CG-B, the strains in CG-A and CG-C were more similar internally. The red labels represent the strains with complete genome sequences through Nanopore MinION long-read sequencing method. A total of 3.4Mbp core sequences were used to calculate the SNP numbers. The SNP numbers between different clades can be obtained by 3.4Mbp times tree scale (0.001) and distance.

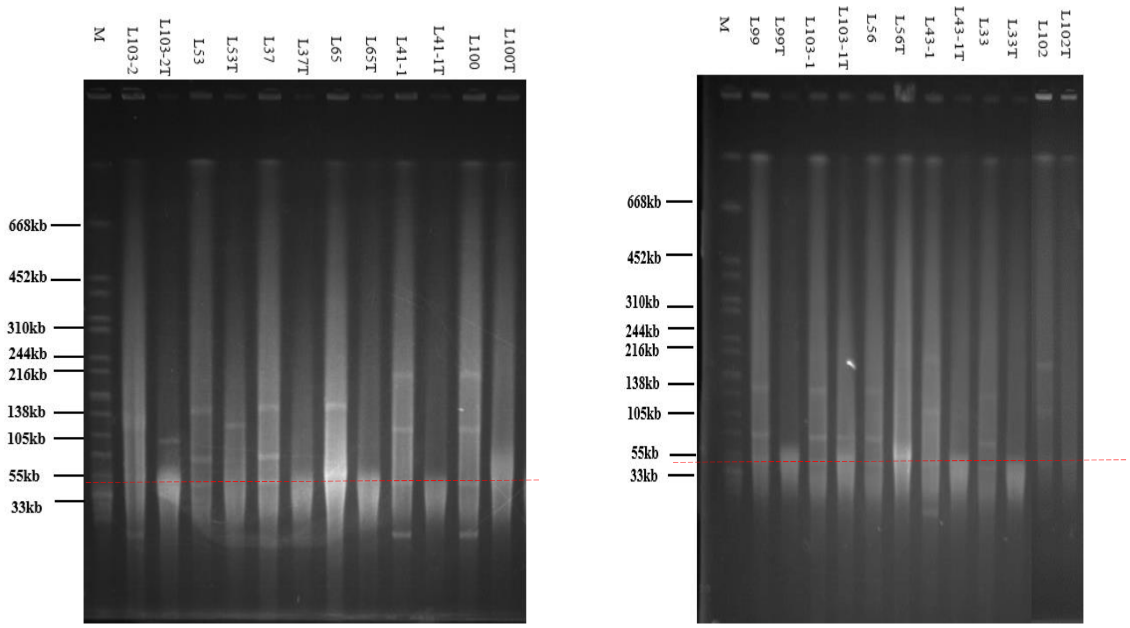

Figure S2. S1-PFGE of *bla*$_{\text{NDM-5}}$ positive isolates and their corresponding transconjugants. Strain IDs with the letter T indicate the matched transconjugants. Other *E.coli* strains can transfer a ca. 45kb plsmid into their transconjugants except the strain L53. M, PFGE type of XbaI digestion of *Salmonella enterica* serotype Braenderup H9812 (molecular marker).

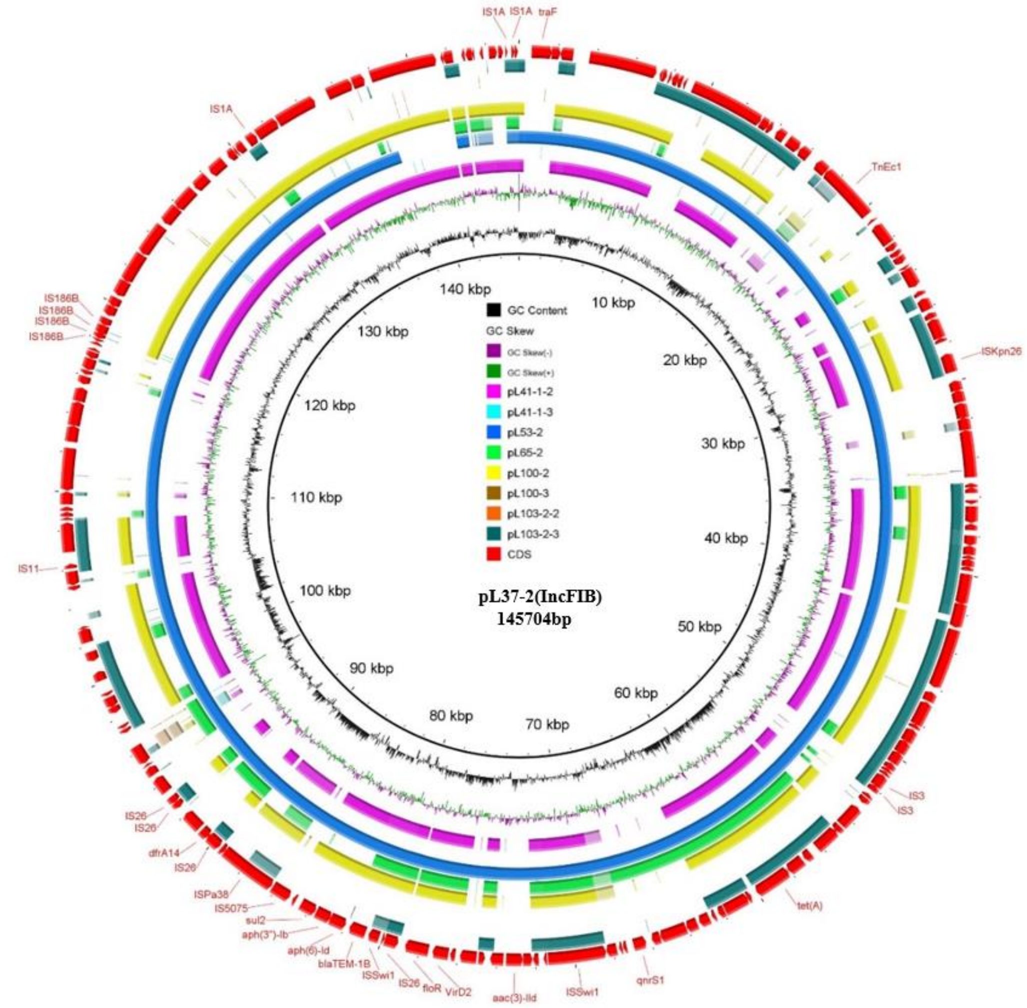

Figure S3. Comparative circular maps of IncFIB plasmids with complete sequences in six *bla*$_{NDM-5}$ positive *E. coli* strains. All plasmids were isolated from the corresponding strains in this study.

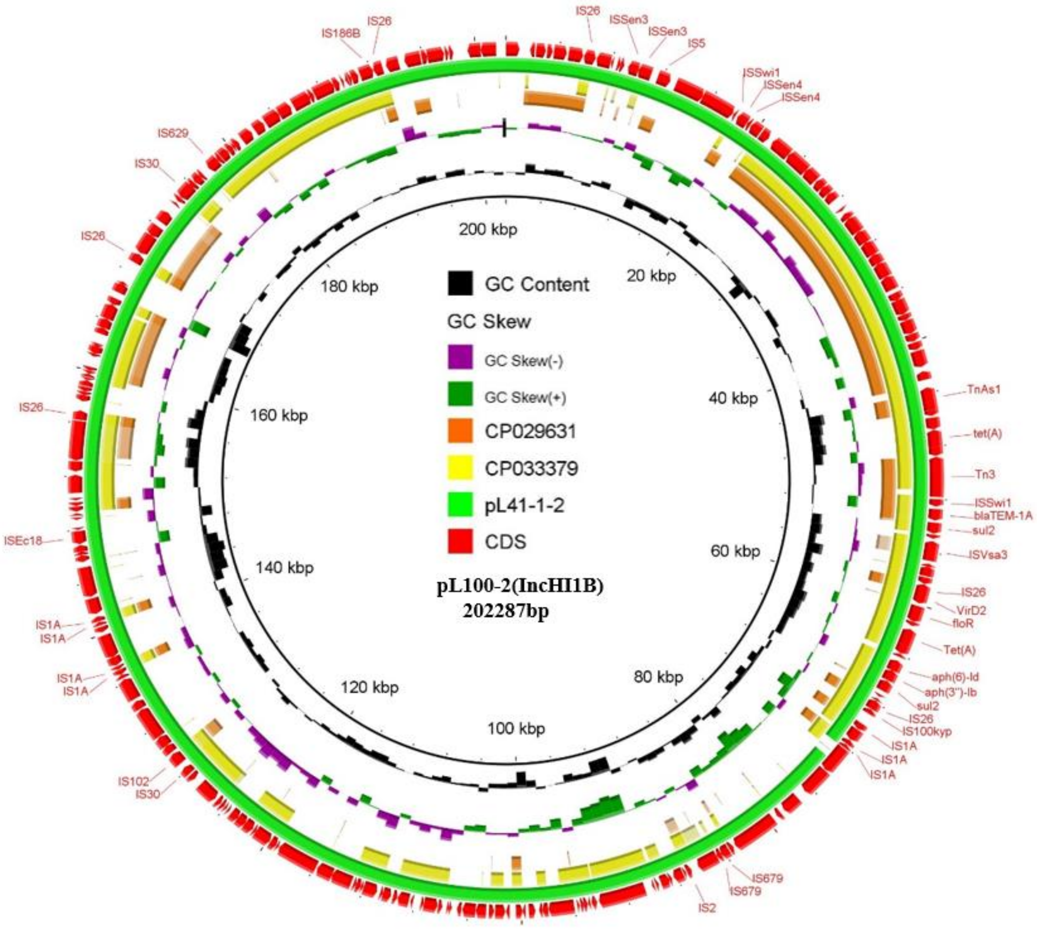

Figure S4. Comparative circular maps of hybrid plasmids containing the IncHI1B replicon. pL100-2(IncFIB, IncHIB, p0111) and pL41-1-2 (IncFIB, IncHIB, p0111) derived from the corresponding strains. CP029631 and CP033379 were plasmids with similar structures retrieved from NCBI database.

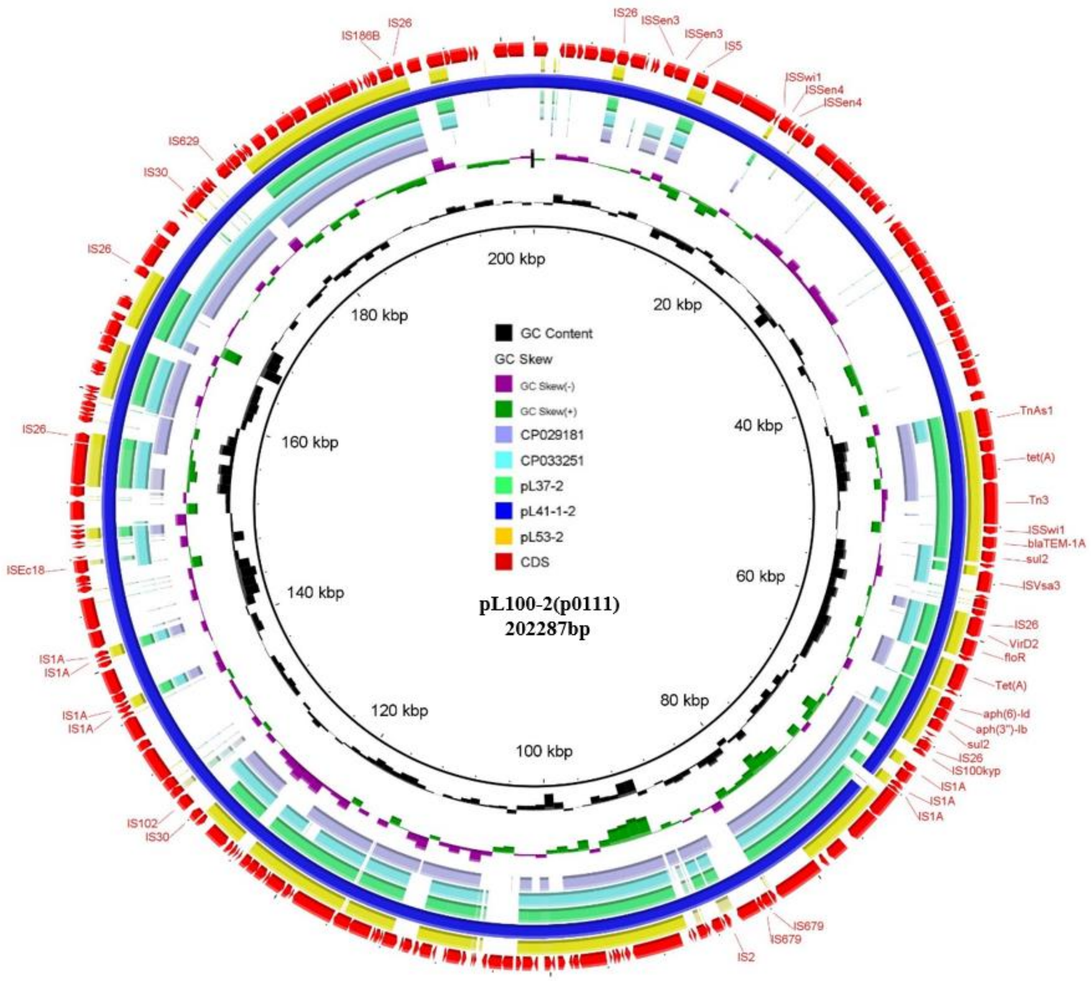

Figure S5. Comparative circular maps of hybrid plasmids containing the p0111 replicon. pL100-2, pL53-2, pL41-1-2 and pL37-2 were isolated from the corresponding strains. CP033251 and CP029181 were plasmid sequences with similar structures from NCBI database.

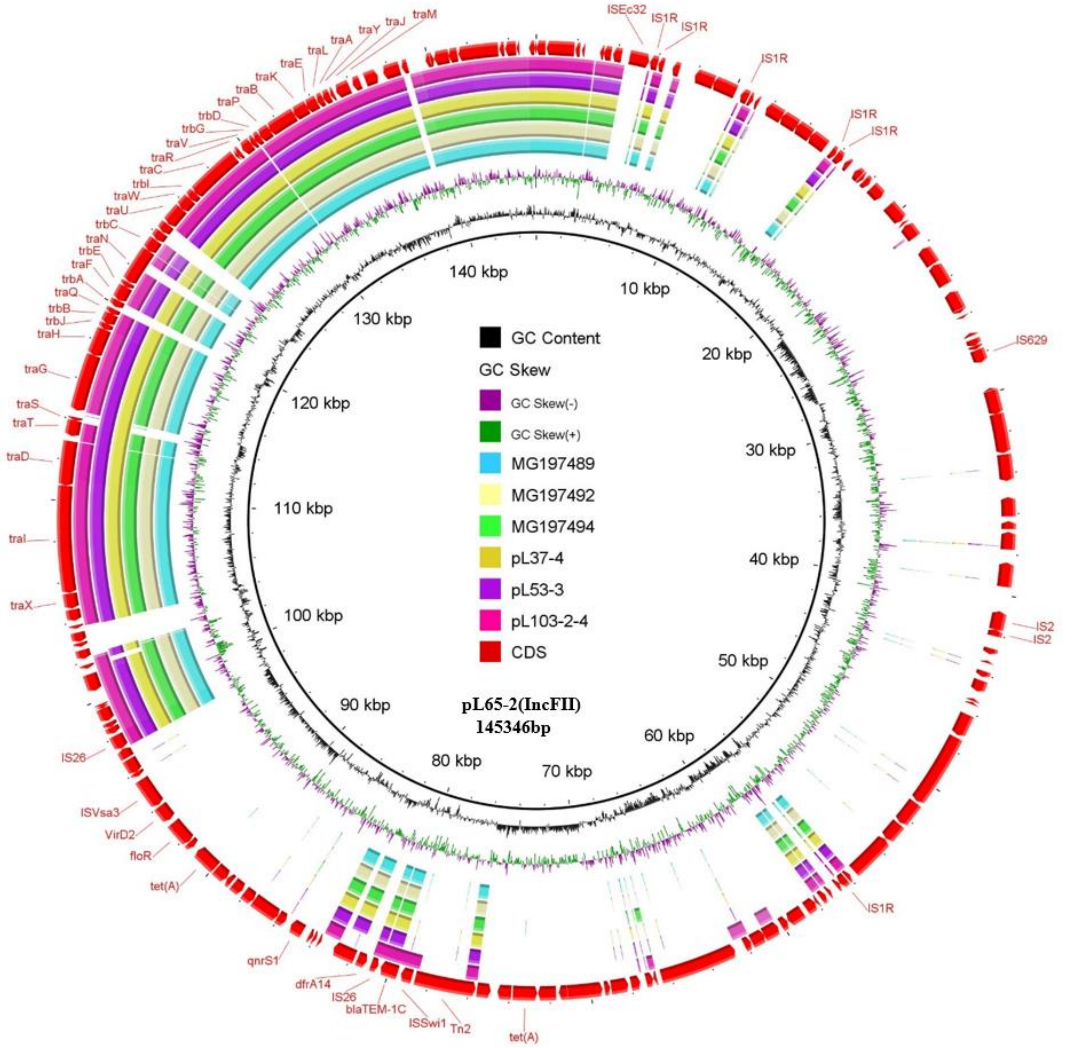

Figure S6. Comparative circular maps of IncFII plasmids. pL65-2 (IncFIB, IncFII), pL37-4, pL53-3 and pL103-2-4 were from the corresponding strains in this study. MG197489, MG197492 and MG197494 were reported plasmid sequences harbouring similar structures in NCBI database.

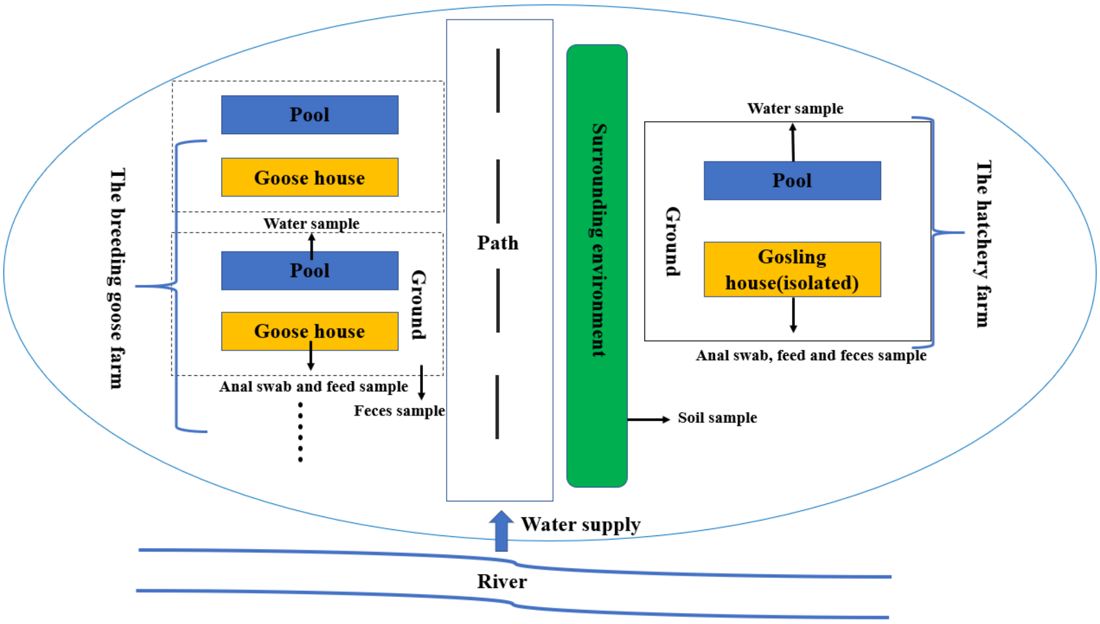

Figure S7. The schematic diagram showing the goose farm overall layout.

Page, A.J., Cummins, C.A., Hunt, M., Wong, V.K., Reuter, S., Holden, M.T., Fookes, M., Falush, D., Keane, J.A., and Parkhill, J. (2015). Roary: rapid large-scale prokaryote pan genome analysis. *Bioinformatics* 31**,** 3691-3693.

Price, M.N., Dehal, P.S., and Arkin, A.P. (2009). FastTree: computing large minimum evolution trees with profiles instead of a distance matrix. *Mol Biol Evol* 26**,** 1641-1650.
